# Host-parasite interactions during *Entamoeba histolytica* infection: an *ex vivo* human intestinal model

**DOI:** 10.3389/fcimb.2026.1870104

**Published:** 2026-07-21

**Authors:** Enrique González-Rivas, Donají González-Jiménez, Jorge Luis De León Rendón, Billy Jiménez Bobadilla, Patricia Morán, Angélica Serrano-Vázquez, Liliana Rojas Velázquez, Martha Elena Zaragoza, Ma. Ángeles Padilla, Eric Hernández, Horacio Pérez-Juárez, Cecilia Ximénez

**Affiliations:** 1 Laboratorio de Inmunología, Unidad de Medicina Experimental, Facultad de Medicina, Universidad Nacional Autónoma de México, Mexico City, Mexico; 2 Servicio de Coloproctología del Hospital General de México “Dr. Eduardo Liceaga”, Mexico City, Mexico

**Keywords:** amebiasis, gene expression, host-parasite interaction, precision-cut intestinal slices (PCIS), qPCR

## Abstract

Amebiasis, caused by the protozoan parasite *Entamoeba histolytica*, remains a significant public health problem in specific endemic regions. Current experimental models have limitations in accurately replicating human pathogenesis, highlighting the need for alternative platforms that preserve tissue complexity and physiological context. This study aimed to evaluate *E. histolytica* infection using an *ex vivo* human precision-cut intestinal slice (PCIS) model by analyzing histological and molecular changes over time (1, 3, 12, 24, and 48 h post-infection). Tissue integrity and parasite invasion were assessed using immunohistochemistry, while the gene expression of parasite virulence factors and host cytokines was quantified by quantitative PCR (qPCR). The PCIS model remained viable and preserved tissue architecture during the first 24 h. Histological analysis revealed progressive epithelial damage and parasite invasion into the connective tissue. Parasite gene expression revealed distinct temporal expression patterns during infection. While the EhGal/GalNAc lectin maintained a relatively stable expression profile throughout the interaction, *Ehambp* and the cysteine proteases (*Ehcp2* and *Ehcp5*) exhibited early activation patterns consistent with roles in parasite adhesion, tissue invasion, and extracellular matrix degradation. In contrast, genes associated with oxidative stress response and parasite survival (*Ehnitro*, *Ehthioredox*, and *Ehdovasa*) displayed increased expression at later stages of infection, suggesting adaptation to the intestinal microenvironment. Host immune response analysis revealed heterogeneous cytokine expression kinetics. Pro-inflammatory mediators, particularly *tnf-α*, *tnf*-β, *il*-2, *il*-4, and *il*-17, showed significant temporal variation during infection, whereas il-10 exhibited progressively increased expression over time. These findings support a dynamic and heterogeneous host immune response during early infection, characterized by distinct temporal expression patterns of pro-inflammatory and regulatory cytokines, rather than a simple Th1-Th2 transition. Overall, these findings demonstrate that the PCIS model is a valuable platform for characterizing the temporal dynamics of host-parasite interactions during early *E. histolytica* infection and provides a physiologically relevant system for investigating the molecular and cellular mechanisms underlying amebiasis.

## 1 Introduction

Amebiasis is an infection caused by the parasitic protozoan *E. histolytica*. Although it is no longer considered a major global public health problem, it remains a significant concern in specific geographic regions, particularly in areas with limited or inadequate public health services. In endemic countries such as Mexico, the prevalence of infection, as assessed by PCR and qPCR-based molecular characterization of *Entamoeba* species, has been reported to be approximately 30% for both *E. histolytica* and *E. dispar* in rural populations (Caballero-Salcedo, et al., 1994; Ramos et al., 2005; CELAC, 2020). Furthermore, recent data on morbidity and mortality associated with this disease are limited. Nevertheless, the epidemiological burden reported by Turkeltaub et al. (2015) highlights its continued impact, with an estimated 10.6 million disability-adjusted life years (DALYs) globally in 2010.

Infection may be asymptomatic, present as invasive intestinal colitis, or manifest in extraintestinal forms, the most common being amebic liver abscess (Walsh and Warren, 1979).

The pathogenesis of amebic intestinal amebiasis (AIA) is a complex and multifactorial process mediated by trophozoites, which invade intestinal tissue through three key steps: (1) adherence, mediated by surface adhesins such as the Gal/GalNAc lectin, which binds to galactose residues on host cell glycoproteins (Haque et al., 2003); (2) cell lysis, induced by virulence factors such as cysteine proteases, which degrade the extracellular matrix, and amebapores, which form pores in host cell membranes and disrupt osmotic balance (Que and Reed, 2000; Becerril, 2023); and (3) immune evasion, in which trophozoites employ mechanisms to counteract host defenses, including degradation of complement components and antibodies (e.g., IgG and IgA), as well as induction of apoptosis in immune cells such as neutrophils (Becerril, 2023).

Despite advances in understanding amebiasis, studying the infection process remains challenging. Animal models (*in vivo*), although useful for evaluating treatments and immune responses, often fail to replicate the full parasitic life cycle and exhibit variable susceptibility due to genetic and dietary factors, limiting their ability to accurately mimic human pathology (Tsutsumi and Shibayama, 2006). Conversely, monolayer cell culture models (*in vitro*) lack the complexity of intact tissue, failing to reproduce tissue architecture, intercellular interactions, and the microenvironmental response.

To overcome these limitations *ex vivo* models have emerged, these preserve tissue viability and three-dimensional structure, enabling a more accurate assessment of host–parasite interactions. The precision-cut intestinal slices (PCIS) model has demonstrated utility in toxicology and metabolism studies (Kanter et al,. 2002; Niu., et al 2013; Pham et al., 2015; Biel et al., 2022; Hung et al., 2023; Li et al., 2023). In the context of amebiasis, it has been validated for use in both liver and intestinal tissues to study pathogenesis, gene expression, and responses to amoebicidal compounds (Carranza Rosales et al,. 2012; Guzmán et al., 2019; Ximénez et al., 2017; Rangel et al., 2019). Unlike *in vivo* models, the PCIS system operates under controlled conditions, reduces the need for animal use, and allows detailed analysis of cellular interactions within a physiologically relevant tissue environment.

Therefore, this study aimed to analyze host–parasite interactions during *E. histolytica* infection using an *ex vivo* PCIS intestinal model at different post-infection times (1, 3, 12, 24, and 48 h), compared with uninfected control slices, through a combined histological, immunological, and molecular approach.

## 2 Materials and methods

### 2.1 Ethical considerations and sample collection

The study protocol was approved by the Research Ethics Committee of the Faculty of Medicine, National Autonomous University of Mexico (UNAM), with approval number FM/DI/068/2023. Intestinal tissue samples were obtained from volunteers at the Coloproctology Service of the General Hospital of Mexico “Dr. Eduardo Liceaga.” Prior to collection, participants undergoing various endoscopic clinical procedures were informed about the study objectives and provided written informed consent for the donation of their intestinal samples. Biopsies corresponded to histologically normal tissue collected from areas adjacent to the affected region in each donor. When the cancerologist indicates the recto sigmoidoscopy and the obtention of tumor biopsy and the normal tissue biopsy, taken 6 cm apart of the tumor lesion, and none of the patients were at this stage of the medical study were submitted to any immunosuppressive treatment. As part of the routine laboratory and clinical diagnosis a microscopic examination was performed and in all the donors the feces microscopic examination for the presence of intestinal parasites were negative.

Four donors were included, and one biopsy per donor was processed. Immediately after collection, samples were placed in cold Krebs–Henseleit (KB) buffer supplemented with antibiotics (ampicillin and gentamicin, 150 and 10 µM, respectively) at 4 °C for transport and preservation (Guzmán et al., 2019).

### 2.2 Preparation of precision-cut intestinal slices

PCIS preparation was based on the method described by Brendel et al. (1990), with modifications by Biel et al. (2022). Samples were processed immediately upon arrival at the laboratory. Each intestinal segment was repeatedly washed with cold KB buffer (4 °C) to remove luminal contents. Superficial adipose tissue and mesentery were carefully removed using a scalpel to preserve the integrity of the muscle layer and mucosa. The cleaned fragments were embedded in 2% agar cylinders to protect the tissue during sectioning. The cylinders were mounted on a vibratome (VT1000S, Leica) to obtain 200 µm-thick transverse sections. Sectioning was performed in oxygenated KB buffer (95% O_2_, 5% CO_2_, 4 °C) to ensure adequate tissue oxygenation. The resulting PCIS were gently transferred to 12-well plates containing 1 mL of DMEM/F12 medium (Invitrogen) and pre-incubated for 1 h at 37 °C to allow tissue stabilization prior to infection assays.

### 2.3 Parasite culture and virulence

Trophozoites of the invasive *E. histolytica* strain HM1: IMSS were cultured under axenic conditions in TYI-S-33 medium (Trypticase–Yeast Extract–Iron–Serum; Diamond et al., 1978), supplemented with 15% bovine serum and 3% vitamins. Cultures were maintained at 37 °C in a humid microaerophilic atmosphere containing 5% CO_2_ for 48 h, corresponding to the logarithmic growth phase, to ensure a viable inoculum for infection assays. To maintain virulence, trophozoites were passed bimonthly through hamster liver (Olivos et al., 2005).

### 2.4 *Ex vivo* infection

Five PCIS from each biopsy were used for infection assays. Each PCIS was inoculated with 1 × 10^5^ *E. histolytica* trophozoites suspended in 1 mL of a 1:1 mixture of DMEM/F12 and TYI-S-33 media. This hybrid medium was supplemented with 2.24 g/L sodium bicarbonate, 50 µg/mL gentamicin, 25 mM glucose, and 1% insulin–transferrin–selenium (ITS) to optimize both tissue and parasite viability. Five uninfected PCIS were used as controls.

Initial co-incubation of infected and control tissues was performed at 37 °C in a humid atmosphere of 95% O_2_ and 5% CO_2_ for 30 min to allow trophozoite adherence to the intestinal tissue. Subsequently, PCIS were maintained under the same conditions with orbital shaking at 50 rpm.

PCIS were collected at different post-infection times (1, 3, 12, 24, and 48 h) to evaluate the progression of invasion. Histological changes and trophozoite localization were assessed by immunohistochemical analysis. All experiments were performed in duplicate.

### 2.5 Immunohistochemical analysis

After incubation, both infected and control PCIS were fixed in 10% formaldehyde and embedded in paraffin. Sections of 10 µm thickness were prepared for each time point (1, 3, 12, 24, and 48 h).

Immunohistochemistry was performed using antibodies against *E. histolytica* calreticulin (Eh-CRT), an immunogenic protein involved in parasite pathogenicity, following the protocol described by Ximénez et al. (2017). Briefly, sections were deparaffinized and subjected to antigen retrieval by heating at 95 °C for 5 min in 10 mM citrate buffer (pH 6.5). Non-specific binding was blocked using 3% PBS-BSA supplemented with 1% bovine serum for 12 h at 4 °C. Samples were then incubated with primary antibodies (1:200 dilution) for 12 h at 4 °C. A goat anti-mouse IgG conjugated to alkaline phosphatase (1:1000 dilution) was used as the secondary antibody. Detection was performed using the NBT/BCIP chromogenic system (Sigma). Finally, sections were counterstained with aqueous eosin, mounted with Glicergel (Sigma), and analyzed under a light microscope.

### 2.6 *In situ* cDNA synthesis and quantification

RNA extraction and cDNA synthesis were performed using an *in-situ* RT-PCR protocol adapted from Nuovo (2007) and Ocadiz et al. (2008, 2009). After incubation at the selected time points (1, 3, 12, 24, and 48 h), both infected and control PCIS were treated with proteinase K (0.5 µg/mL) for 30 min at room temperature, followed by DNase I digestion (1 U/sample) for 48 h at room temperature. Samples were then washed with DEPC-treated water. Reverse transcription was performed using SuperScript II RT (Invitrogen) according to the manufacturer’s instructions. Briefly, 25 µL of reaction mixture containing oligo(dT), dNTPs (10 mM), 5× First-Strand buffer, DTT (0.1 M), RNase inhibitor (40 U/µL), and reverse transcriptase (100 U) was added to each sample. The mixture was incubated at 42 °C for 2 h. The synthesized cDNA was quantified by spectrophotometry (260/280 nm) and adjusted to a concentration of 15 ng/µL for qPCR analysis.

### 2.7 Gene expression analysis by quantitative PCR

The expression of *E. histolytica* genes associated with pathogenicity (*EhGal/GalNAc lectin*, *Ehdovasa*, *Ehthioredoxin*, *Ehnitro*, *Ehcp-2*, *Ehcp-5*, *Ehambp*, *Ehhypo3*, *Ehhypo1*, *Ehhypo6*, with *Ehα-actin* as the reference gene) and host immune response genes (*tnf-α*, *ifn-α*, *il-4*, *il-2*, *il-8*, *il-10*, *il-17a*, *tnf-β*, with human *β-actin* as the reference gene) was evaluated using qPCR on a StepOne™ real-time thermocycler (Applied Biosystems).

Each reaction had a final volume of 20 µL, containing 10 µL of 2× SYBR^®^ Green master mix (QuantiTect SYBR Green Kit, Qiagen), 0.2 µM of each forward and reverse primer, and 2 µL of cDNA (approximately 15 ng/µL). The volume was adjusted with nuclease-free water. Primer sequences are listed in supplementary **Supplementary Table 1**.

The amplification protocol consisted of an initial denaturation at 95 °C for 15 min, followed by 40 cycles of denaturation (94 °C for 15 s), annealing (55 °C for 30 s for *E. histolytica* genes or 60 °C for 15 s for human genes), and extension (75 °C for 30 s). A melting curve analysis was performed to verify product specificity.

All reactions were performed in triplicate. Relative gene expression was calculated using the 2^-^ΔΔCt method (Livak and Schmittgen, 2008), normalized against the corresponding reference genes, obtaining the quantification relative (QR).

### 2.8 Statistical analysis

Statistical analyses were performed using Jamovi software (version 2.7.24). Data distribution was assessed using the Shapiro-Wilk test, and homogeneity of variances was evaluated using Levene’s test. When necessary, gene expression data were log10-transformed to satisfy the assumptions of parametric analyses. Datasets meeting the assumptions of normality and homogeneity of variances were analyzed using one-way ANOVA, followed by Tukey’s *post hoc* test. When normality was achieved but homogeneity of variances was not met, Welch’s ANOVA followed by the Games-Howell *post hoc* test was applied. Effect sizes were estimated to assess the magnitude of temporal differences in gene expression. Datasets that did not satisfy the assumptions of parametric analyses after log10 transformation were analyzed using the non-parametric Kruskal–Wallis test, followed by Dunn’s multiple comparisons test with Bonferroni adjustment. Gene expression data were graphically represented as box plots displaying the median, interquartile range, and minimum-maximum values, with overlaid individual data points representing independent biological samples to illustrate biological variability. Statistical significance was established at p < 0.05.

## 3 Results

### 3.1 Tissue viability and immunohistochemical analysis of infection in the *ex vivo* model

Intestinal tissue samples were obtained from four donors (50% male, 50% female; mean age 45.75 ± 10.53 years). All patients had lower rectal tumors; however, the biopsies corresponded to histologically normal tissue collected from areas adjacent to the affected region in each donor.

**Figure 1:**
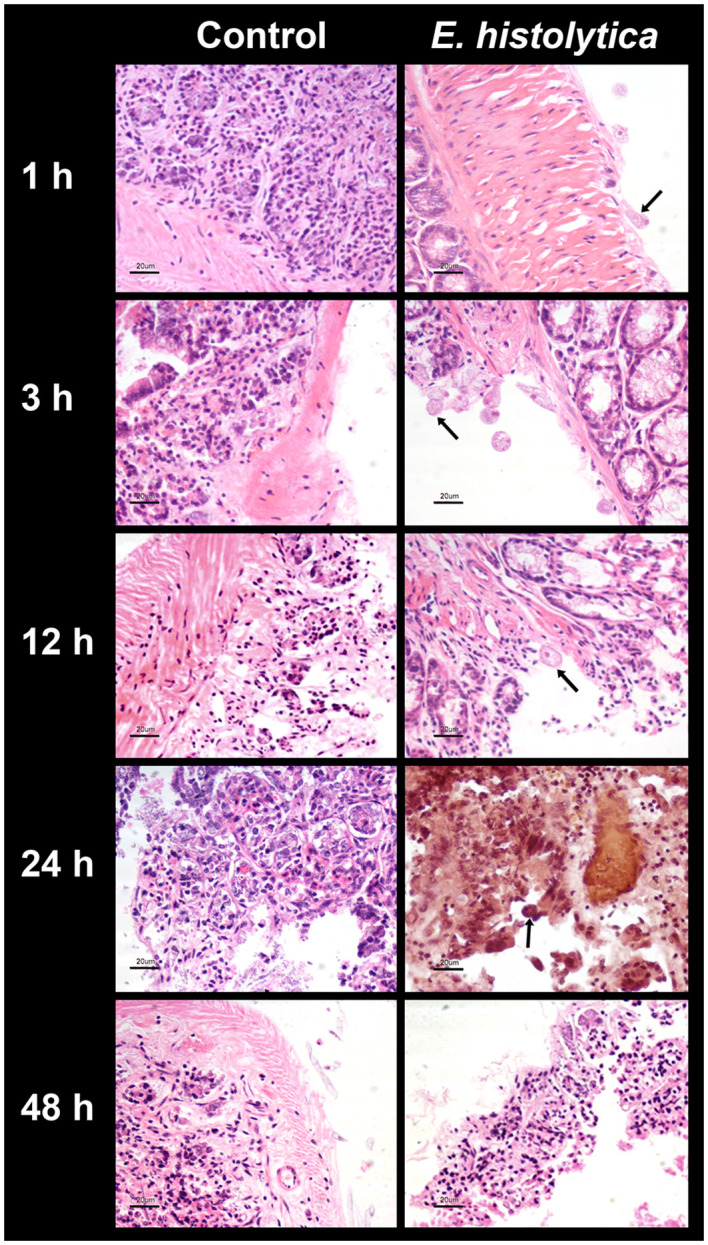
Immunodetection of calreticulin (CRT) in human intestinal PCIS infected *ex vivo* with *E. histolytica* trophozoites at 1, 3, 12, 24, and 48 h. Control samples show intact epithelium until 24h. Infection leads to progressive epithelial disruption, heterogeneous CRT distribution, and intense staining in damaged areas closely associated with trophozoites. Trofozoitos señalados. Scale bar = 20 µm.

Microscopic analysis of PCIS confirmed the integrity and viability of the intestinal tissue, control slices maintained well-preserved intestinal architecture, with intact crypts of Lieberkühn and enterocytes at 1, 3, 12, 24, and 48 h. No bacterial component was detected in any samples. Although, overall tissue integrity was preserved, the mucosal layer was no longer observable after the first hour post infection (**Figure 1**).

In contrast, infected PCIS showed a progressive deterioration of tissue integrity at 1 h post-infection, the presence of *E. histolytica* trophozoites was detected using anti-calreticulin (anti-EhCRT) antibodies, anti-EhCRT immunostaining showed a weak signal, with no clear evidence of trophozoites associated with the tissue. At 3 h post-infection, the tissue exhibited a loss of epithelial cell integrity with connective tissue becoming the predominant component, at this time point, identification of lysed trophozoites, suggesting the onset of parasite–tissue interaction (**Figure 1**).

At 12 h after infection several enterocytes were detached from the mucosal layer, accompanied by an increasement in the number of trophozoites in the connective tissue, indicating progression of the infection process, and a process of adaptation of trophozoites, however, at 24 and 48 h, the parasitic load decreased, along with marked epithelial degradation (**Figure 1**).

### 3.2 Expression profile of *E. histolytica* virulence and survival-associated genes

**Figure 2:**
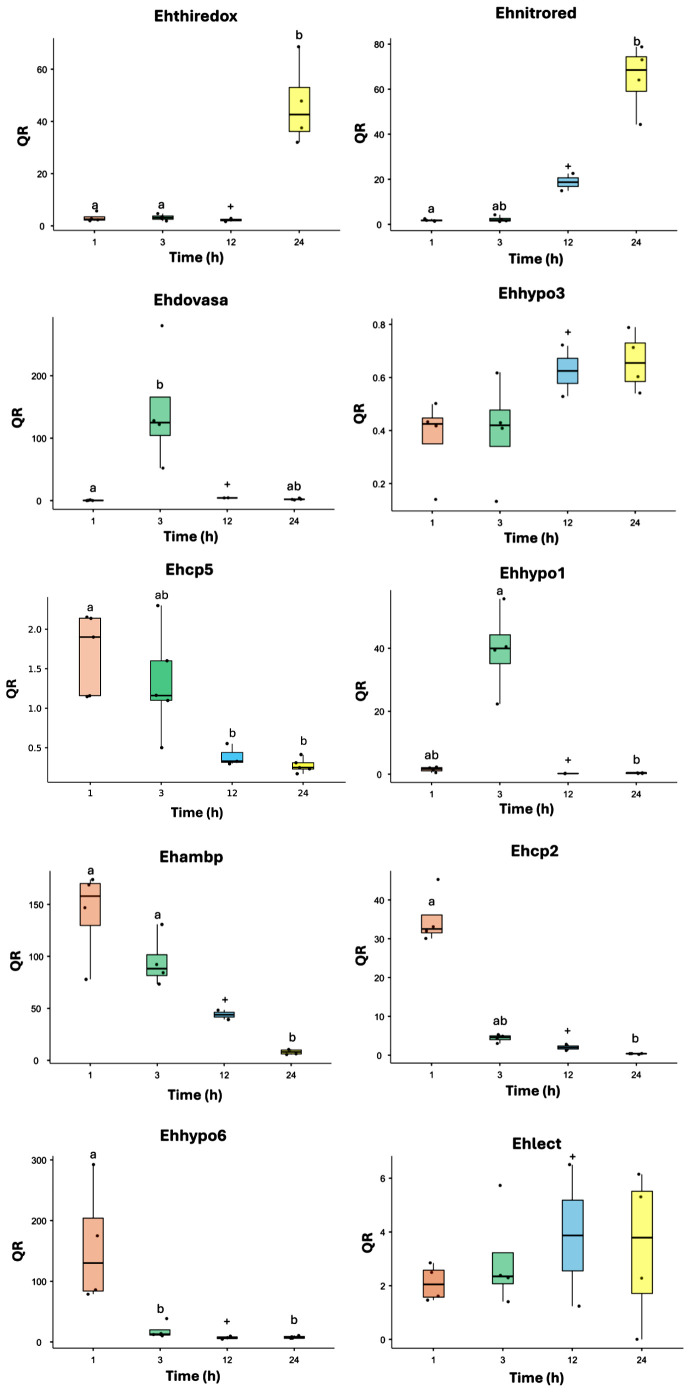
Expression profile of *E. histolytica* virulence-associated genes during host–parasite interaction. Relative expression levels of parasite genes were evaluated at 1, 3, 12, and 24 h post-interaction. Genes analyzed include pathogenicity-associated genes (*Ehlect*, *Ehambp*, *Ehcp2*, *Ehcp5*), oxidative stress response genes (*Ehnitrored*, *Ehthiredox*), hypothetical proteins (*Ehhypo1*, *Ehhypo3*, *Ehhypo6*), and regulatory genes (*Ehdovasa*). Data are presented as box plots showing the median, interquartile range, and minimum-maximum values, with individual data points overlaid to illustrate biological variability. Different letters (a, b, c) indicate statistically significant differences between groups (p < 0.05), based on Tukey, Games-Howell, or Dunn *post hoc* tests, as appropriate. No significant differences were observed for genes without letter annotations. (+) The samples collected at 12 h when n = 2 were included for visualization purposes only and were excluded from the statistical analyses due to an insufficient sample size.

The expression profiles of *E. histolytica* genes revealed distinct temporal patterns during the host-parasite interaction (**Figure 2**). Descriptive statistics for parasite gene expression across all post-infection time points are summarized in **Supplementary Table 2**. Genes associated with parasite adhesion exhibited different dynamics: while the *EhGal/GalNAc lectin* maintained a relatively stable expression pattern without significant temporal differences, *Ehambp* showed its highest expression during the early stages of infection, followed by a progressive decline, reaching significantly lower levels at later time points. Similarly, genes involved in tissue invasion and damage displayed an early activation pattern. Both *Ehcp2* and *Ehcp5* exhibited their highest expression during the initial stages of infection, followed by reduced expression as the interaction progressed. In contrast, genes associated with parasite survival and adaptation to the host environment showed delayed activation. *Ehnitrored* and *Ehthioredox* exhibited increased expression during the later stages of infection. Hypothetical proteins displayed heterogeneous expression profiles: *Ehhypo1* showed a transient peak in expression followed by a marked decline, whereas *Ehhypo6* gradually decreased over time. In contrast, *Ehhypo3* maintained a relatively stable expression profile throughout the evaluated time points without significant temporal variation. The regulatory gene *Ehdovasa* exhibited a transient increase in expression during the early stages of infection, followed by reduced expression at later time points. One-way ANOVA and Welch’s ANOVA revealed significant differences across post-infection time points for *ehcp5* (F = 13.7, p < 0.004), *Ehambp* (F = 21.4, p < 0.001), and *Ehhypo6* (F = 31.6, p < 0.001). In contrast, no significant differences were observed for *EhGal/GalNAc lectin* (F = 0.451, p = 0.651) or *Ehhypo3* (F = 3.88, p = 0.061) (**Table 1**). For datasets that did not satisfy the assumptions of parametric analyses after data transformation, Kruskal–Wallis analysis showed significant temporal differences in several *E. histolytica* genes. Significant differences were observed for *Ehcp2* (χ² = 9.85, p = 0.007), *Ehnitrored* (χ² = 7.42, p = 0.024), *Ehthioredox* (χ² = 7.42, p = 0.024), *Ehhypo1* (χ² = 9.88, p = 0.007), and *Ehdovasa* (χ² = 9.85, p = 0.007), indicating time-dependent variation in their expression profiles during infection (**Table 2**). Pairwise *post hoc* comparisons using Tukey’s, Games-Howell, or Dunn’s tests with Bonferroni adjustment, as appropriate, identified significant differences between specific post-infection time points (**Supplementary Tables 4**, **S5**). Due to the limited number of biological replicates at 12 h (when n = 2), these data were excluded from the inferential statistical analyses and are presented descriptively only to illustrate temporal expression trends.

**Table 1:** One-way ANOVA and Welch's ANOVA results for *E. histolytica* gene expression across post-infection time points.

Gene	F (df)	p-value	η²p
Ehlect	F (2, 9) = 0.451	0.651	0.091
Ehambp	F (2, 9) = 21.4	**<0.001**	0.826
Ehcp5	F (3, 6.15) = 13.7	**0.004**	–
Ehhypo3	F (2, 9) = 3.88	0.061	0.463
Ehhypo6 (log10)	F (2, 9) = 31.6	**<0.001**	0.875

The 12 h group (when n = 2) was excluded from statistical analyses due to insufficient sample size.

Bold values indicating the statisticaly significant differences between groups.

**Table 2:** Kruskal–Wallis test results for *E. histolytica* gene expression across post-infection time points.

Gene	χ² (df)	p-value	ϵ²
Ehcp2	χ² (2) = 9.85	**0.007**	0.895
Ehnitrored	χ² (2) = 7.42	**0.024**	0.675
Ehthiredox	χ² (2) = 7.42	**0.024**	0.675
Ehhypo1	χ² (2) = 9.88	**0.007**	0.898
Ehdovasa	χ² (2) = 9.85	**0.007**	0.895

The 12 h group (n = 2) was excluded from statistical analyses due to insufficient sample size.

Bold values indicating the statisticaly significant differences between groups.

### 3.3 Gene expression analysis of host immune response

**Figure 3:**
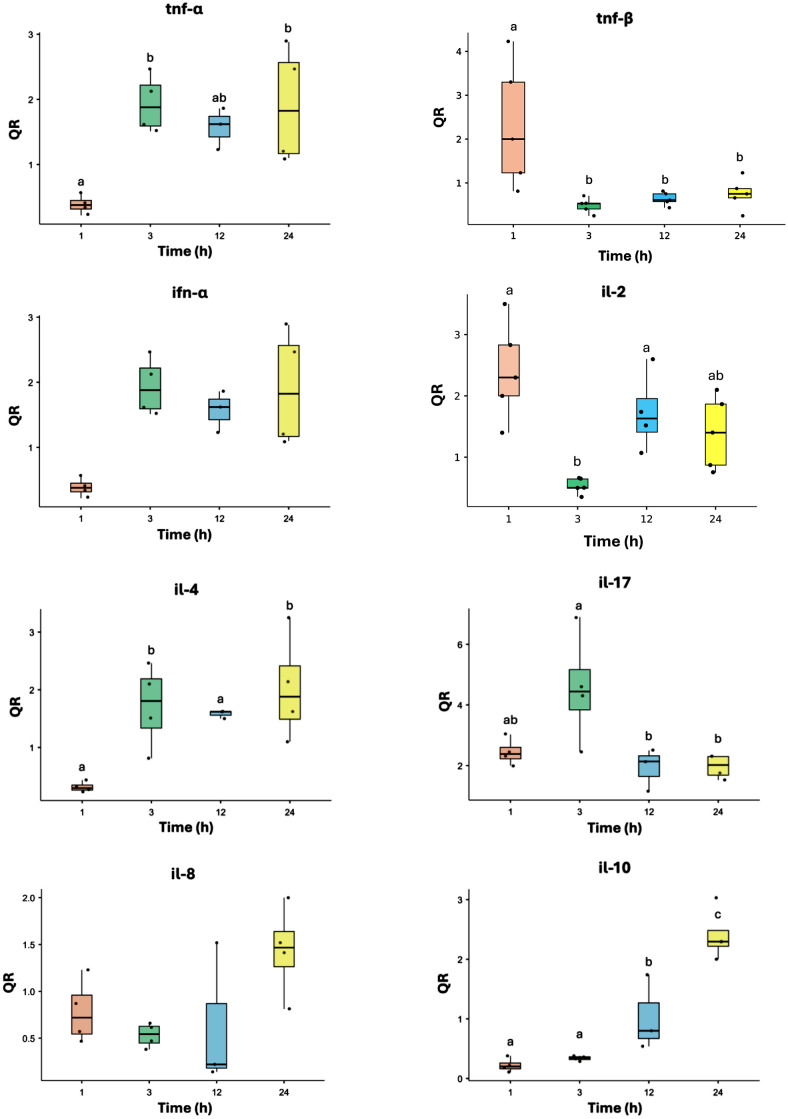
Relative gene expression of immune response mediators in human intestinal PCIS infected ex vivo with *Entamoeba histolytica* trophozoites at 1, 3, 12, 24, and 48 h. Cytokine mRNA levels were quantified by qPCR and expressed as relative quantification (QR) values. Data are presented as box plots showing the median, interquartile range, and minimum-maximum values, with individual data points overlaid to illustrate biological variability. Each point represents an independent biological sample. Different letters (a, b, c) indicate statistically significant differences between time points (p < 0.05), based on Tukey and Games-Howell *post hoc* tests, as appropriate. No significant differences were observed for genes without letter annotations.

The expression profiles of host immune response genes revealed dynamic and time-dependent cytokine kinetics during infection (**Figure 3**). Descriptive statistics for host immune response genes are provided in **Supplementary Table 3**. Pro-inflammatory cytokines showed an early activation pattern: *tnf-α*, *il-2*, and *il-17* reached their highest expression levels at 3 h post-infection, followed by a decrease at later time points (12–24 h). Similarly, tnf-β exhibited significant temporal variation, supporting its involvement in the early inflammatory response. In contrast, showed delayed activation patterns: *il-10* exhibited a progressive increase throughout the infection process, reaching its highest expression at 24 h. Likewise, *ifn-α* showed an increasing trend at later time points, although considerable inter-individual variability was observed, and *il-8* displayed a distinct kinetic profile, with reduced expression at intermediate time points followed by increased expression at 24 h.

Parametric analyses (one-way ANOVA or Welch’s ANOVA, as appropriate) of host immune response genes revealed significant temporal differences in cytokine genes expression. ANOVA tests showed significant effects of infection time on *il-10* log 10 (F = 31.6, p <0.001), *il-2* (F = 8.58, p = 0.001), *il-4* (F = 6.04, p = 0.011), *tnf-α* (F = 7.06, p = 0.007), *tnf-β* (χ² = 7.69, p = 0.002) and *il-17* (F = 5.51, p = 0.015). In contrast, *ifn-α* log 10 (F = 3.55, p = 0.051) and *il-8* (F = 3.08, p = 0.072) did not reach statistically significant across time point (**Table 3**). *Post hoc* multiple comparisons (Tukey/Games-Howell) identified pairwise differences between time points (**Supplementary Table 6**).

**Table 3:** One-way ANOVA and Welch's ANOVA results for host cytokine gene expression across post-infection time points.

Gene	F (df)	p-value	η²p
tnf-α	F (3,11) = 7.06	**0.007**	–
tnf-β (log10)	F (3,16) = 7.69	**0.002**	0.591
il-8	F (3,11) = 3.08	0.072	0.457
il-2	F (3,15) = 8.58	**0.001**	0.632
il-4	F (3,11) = 6.04	**0.011**	–
il-17	F (3,11) = 5.51	**0.015**	0.600
il-10 (log10)	F (3,11) = 31.6	**<0.001**	0.896
ifn-α (log10)	F (3,11) = 3.55	0.051	0.492

Bold values indicating the statisticaly significant differences between groups.

## 4 Discussions

The present study provides new insights into parasite-host interactions during *E. histolytica* infection using an *ex vivo* intestinal model. This model is particularly valuable, as it allows direct observation of pathological processes in a physiologically relevant human tissue system and provides a platform to analyze early host-parasite interactions, especially during the initial hours of infection.

Control PCIS preserved the structure and components of human intestinal tissue, providing a reference for assessing infection-induced changes. In contrast, infected PCIS exhibited alterations consistent with the onset of parasite-host interaction. These results demonstrate that trophozoites induce progressive tissue damage. The observed enterocyte detachment at 12 h and epithelial degradation at 48 h suggest that the parasite not only adheres but also actively triggers mechanisms of invasion and host tissue destruction, as reported in other models (Bansal et al., 2009; Mortimer and Chadee, 2010). Furthermore, submucosal invasion was evident; the presence of trophozoites within the connective tissue confirms the parasite’s ability to penetrate beyond the mucosal layer, a hallmark of invasive intestinal amebiasis (Keene et al., 1990; Que and Reed, 2000). These findings support the suitability of the PCIS model for studying early invasion events preceding ulcer formation (Becerril, 2023).

Phagocytosis of host cells, such as lymphocytes or erythrocytes, represents a strategy for nutrient acquisition and evasion of host immune responses (Mortimer and Chadee, 2010). The ability of *E. histolytica* trophozoites to phagocytose immune cells to prevent their elimination has been documented *in vitro*, and our findings are consistent with these observations in a human tissue context. Moreover, the presence of trophozoites invading connective tissue and containing phagocytosed immune cells at later stages provides direct evidence consistent with previous studies in animal, cellular models and explants of liver tissue (Tsutsumi and Shibayama, 2006; Ximénez et al., 2017).

Overexpression of the EhGal/GalNAclectin and EhCP2 during early invasion stages is well documented. The Gal/GalNAc lectin is essential for adhesion, while cysteine proteases are critical for extracellular matrix degradation (Frederick and Petri, 2005). Consistent with these roles, the early overexpression of these genes suggests that adhesion and proteolysis are fast activated processes that determine the success of parasite invasion (Edman et al.,1990; Bansal et al., 2009). Although, this finding contrasts from some *in vitro* studies (Que and Reed, 2000), it aligns with observations from human explant models analyzing parasite transcriptomic responses (Naiyer et al., 2019; Thibeaux et al., 2013).

The expression of cysteine protease genes (*Ehcp2* and *Ehcp5*) showed a progressive decline following the early peak expression (**Figure 2**). This pattern differs from that reported in human precision cut liver slices (PCLS), where *Ehcp2* expression increased steadily up to 24 h, whereas in *E. dispar* overexpression was observed only during the first hour (Ximénez et al., 2017). Tissue type may influence the gene expression dynamics, as higher expression levels of CP2 and CP5 have been reported in amoebic liver abscess and genital cutaneous amoebiasis (González et al., 2020).

The early expression profile of cysteine proteases observed in our model may reflect their role in extracellular matrix degradation. Together with CP1 and CP3, these enzymes degrade fibronectin, laminin, and collagen, facilitating cell detachment, inducing apoptosis, and degrading immunoglobulins, cytokines, and defensins (Becerril, 2023).

Taken together, our results suggest that CP2 and CP5 act primarily during the early phase of *ex vivo* intestinal infection, while their subsequent downregulation may reflect regulatory mechanisms or a shift toward other virulence factors. This is consistent with the dynamic and multifactorial nature of *E. histolytica* virulence and may explain the sustained expression of the *Ehambp* gene.

The subsequent activation of genes such as *Ehnitro* and *Ehthioredoxin* suggests adaptation and survival mechanisms in response to the intestinal microenvironment. Thioredoxin showed its highest expression at 1 h (**Figure 2**), indicating early activation of antioxidant defenses. This may reflect the need for trophozoites to counteract oxidative stress and favoring parasite persistence (Ajuebor et al., 2002; Weber et al., 2016), through modulation of cytokines and chemokines (IL-1, IL-8, IL-6) (Tsutsurni, 1994; Pacheco et al., 2011).

In the present human model, *il-10* showed its highest expression at 48 h (**Figure 3**). This differs from hamster models of amoebic liver abscess, where *il-10* highest expression is during the early stages of infection and declines rapidly (Carranza et al., 2012), suggesting species-specific regulatory dynamics. In human PCLS, *E. dispar* induces *il-10* at 48 h after infection, whereas *E. histolytica* shows minimal expression at the same period (Ximénez et al., 2017). In our model, sustained *il-10* expression may reflect host attempts to limit tissue damage allowing parasite persistence.

IL-17A showed peak expression at 3 h followed by a marked decline at 48 h (**Figure 3**). This differs from PCLS models, where early overexpression occurs at 1 h (Ximénez et al., 2017). In murine models, IL-17A is not essential for invasion but contributes to parasite persistence by modulating Th1 responses (Deloer et al., 2017).

In our model, the decline in IL-17A coincided with increased IL-4 and IL-10 expression (**Figure 3**), suggesting a shift toward a Th2-type response. Despite this shift, parasite virulence gene expression decreased at later stages, likely associated with reduced parasite load. Early increases in TNF-β, TNF-α, and IL-2 were followed by a decline of expression at later time points, indicating a transient Th1 response. This dual pattern may reflect a balance between early infection control and later regulation to limit tissue damage.

IL-8 exhibited a kinetic pattern opposite TNF-α, with high expression levels at later stages. Both cytokines are associated with inflammation and parasite invasion (Blázquez et al., 2006; Galván et al., 2008). This differential expression suggests that cytokine polarization may not directly drive parasite migration in this model. In animal models, IL-8 remains elevated during chronic infection and granuloma formation (Pacheco et al., 2011), indicating a role in sustained immune responses.

*ifn-α* showed increased expression at 48 h, whereas *tnf-α* peaked early and declined overtime. This contrasts with hamster models, where both cytokines decrease as infection progresses (Pacheco et al., 2011), further highlighting species-specific immune dynamics.

Taken together, these findings indicate that the immune response in this human model is dynamic than the alternative *in vitro* or *in vivo* animal models. Th1-associated cytokines seem to dominate during the early phase of infection followed by a later phase characterized by increased *il-10* and *il-4* expression, suggesting a shift toward a Th2-type response (Campbell and Chadee, 1997). This transition may have a dual role: limiting host tissue damage and consequently allowing the persistence of parasite infection through the reduction of its virulence mechanism and increasing the parasite adaptive strategies.

However, the interpretation of our findings requires caution due to several methodological limitations. The main limitation is the small sample size (4), which increases the susceptibility of the results to inter-individual biological variability. Nevertheless, robust statistical approaches suitable for small sample sizes were applied when n was equal to or greater than 3, allowing the identification of statistically significant differences.

Additionally, the model itself has inherent limitations related to the origin of the tissue samples. Although histologically normal adjacent tissue was consistently used, the underlying clinical condition of each donor may influence the immunological and structural responses of the tissue. It is important to mention that the samples used in this work correspond to: Cancer biopsies that have not received any type of previous treatment (such as surgery, chemotherapy, radiotherapy or immunotherapy) are called virgin biopsies or are also described as untreated tumor samples. Using these tissues as controls can complicate interpretation, as they may not represent a truly healthy reference expression. Ideally, screenings should come from people without cancer, although practical and ethical constraints often make this difficult. However, when we determine the differential expression of the different genes by qPCR, expressing this as: Relative quantification (QR) is usually determined by the ΔΔCt method (comparison of threshold cycles), using stable reference genes to normalize and comparing it with a calibrating sample (control) which in our case is the intestinal tissue not exposed to the parasite. So, background noise would be limited.

Another inherent limitation is the restricted long-term viability of the *ex vivo* tissue, which may introduce bias at later stages of infection due to progressive tissue degradation. Future studies will focus on optimizing culture and incubation conditions to improve tissue viability over extended periods.

## 5 Conclusions

The *ex vivo* human PCIS model reproduced key histological changes induced by the parasite infection and enabled the analysis of the dynamics of parasite-tissue interactions, during the early stages of human infection.

The temporal expression patterns of parasite virulence-associated genes support distinct roles during infection establishment. While the EhGal/GalNAc lectin maintained a relatively stable expression profile, *Ehambp*, *Ehcp2*, and *Ehcp5* exhibited early activation patterns consistent with their involvement in adhesion, tissue invasion, and mucosal degradation, highlighting the importance of these processes for parasite establishment.

The subsequent activation of genes associated with parasite protection and survival (*Ehnitrored* and *Ehthioredox*) suggests that the parasite responds to oxidative stress induced by the host tissue, which may contribute to its survival.

The transient expression pattern of *Ehdovasa*, an immunodominant antigen, suggests a potential role during the early stages of infection and may be associated with mechanisms involved in parasite adaptation and host–parasite interactions.

Regarding the host immune response, heterogeneous cytokine kinetics were observed. Pro-inflammatory mediators, particularly *tnf-α*, *tnf-β*, *il-2*, *il-4*, and *il-17*, exhibited significant temporal variation during infection, whereas *il-10* showed a progressive increase over time. *ifn-α* and *il-8* displayed temporal trends but did not reach statistical significance. Overall, these findings support a dynamic balance between early inflammatory responses and subsequent regulatory mechanisms.

Overall, the PCIS model represents a valuable platform for translational research, as it recapitulates key events of human amebiasis and enables the study of host-parasite interactions in a physiologically relevant context.

This model can be used to evaluate the efficacy of therapeutic compounds in a living tissue environment, analyze variability among *E. histolytica* strains, and investigate immune responses in tissues with different genetic backgrounds, thereby supporting its application in translational research.

The PCIS model may be applied to screen amoebicidal compounds, both natural and synthetic, allowing a more accurate assessment of therapeutic compounds host cell toxicity, and potentially accelerating the development of new treatments.

Although the PCIS model represents a promising platform for translational studies of amebiasis, additional validation using larger sample sizes, extended tissue viability, and a broader range of biological conditions will be necessary to further support its application in therapeutic screening, strain variability analysis, and personalized host-parasite interaction studies.

## Data Availability

The original contributions presented in the study are included in the article/**Supplementary Material**. Further inquiries can be directed to the corresponding authors.
