# Supplementary material for: Host-parasite interactions during Entamoeba histolytica infection: an ex vivo human intestinal model
Source: Front Cell Infect Microbiol. 2026 Jul 21;16:1870104. doi: 10.3389/fcimb.2026.1870104 (PMC13433179; doi:10.3389/fcimb.2026.1870104)
Supplement: Supplementary file 1 [file DataSheet1.pdf]

Supplementary table S1. Primers and their sequences that were used in this study.

Gene	Access number	Product size	Forward 5' - 3'	Reverse 5' - 3'
Homo sapiens sapiens genes				
**tnf-α**	**X02910.1**	**107**	**GCCCTACTATTCAGTGGCGA**	**GAGCTTCTTCCCACCCACAA**
**ifn-α**	**NM_024013**	**182**	**CTTGTGCCTGGGAGGTTGTC**	**TAGCAGGGGTGAGAGTCTTTG**
**il-4**	**NM_000589**	**194**	**GTGCACCGAGTTGACCGTAA**	**TGTCGAGCCGTTTCAGGAAT**
**il-2**	**NM_000586.4**	**131**	**TGTCACAAACAGTGCACCTACT**	**GCATCCTGGTGAGTTTGGGA**
**il-8**	**NM_000584.3**	**183**	**GGTGCAGTTTTGCCAAGGAG**	**TTCCTTGGGGTCCAGACAGA**
**il-10**	**NM_000572.2**	**135**	**AAGACCCAGACATCAAGGCG**	**AGGCATTCTTCACCTGCTCC**
**il-17A**	**NM_002190.2**	**133**	**CCTTGGAATCTCCACCGCAA**	**GCTGGATGGGGACAGAGTTC**
**tnf-β**	**X01393.1**	**155**	**TCTCCCCATTCTGCCTCCATT**	**GGATGGTTCAGGGAGTGTGGG**
**Reference gene Actin -β**	**NM_001101.3**	**166**	**CTCACCATGGATGATGATATC**	**AGGAATCCTTCTGACCCATGC**
Entamoeba histolytica genes				
**Electa gal/galnac**	**AF337950.1**	**281**	**ACCAGTGAATGGAGCATGTGT**	**TTGTGCATTCGCCTTCTCCT**
**Variable surface immunodominant antigen (Ehdovasa)**	**XM_649416**	**188**	**ATACATTTAAAATAGGATCA**	**GCATCTTCCAAACTTCTAAT**
**Thioredoxin reductase (Ehtioredxr)**	**XM_650656.2**	**180**	**TTAATGGTAGTTGGGGA**	**TAATTCTACTAATTCAGA**
**Protein from the nitroreductase family (Ehnitro)**	**XM_651283.1**	**156**	**TGTATGGCTAGGAGTATA**	**AAACGAGTTGCATCAAAT**
**Cysteine Protease 2 (Ehcp-2)**	**XM_645550**	**115**	**ATCCAAGCACCAGAATCAGT**	**TTCCTTCAAGAGCTGCAAGT**
**Cysteine protease 5 (Ehcp-5)**	**XM_645845.2**	**255**	**GTTGCCGCTGCTATTGATGC**	**ACCACTGGATAAGCAGC**
**Hypothetical Protein 3 (Ehhypo-3)**	**XM_649355.2**	**244**	**TTGTCGTTTATTCTCACCAAA**	**AAAGATTTAACTTACCTGGAA**
**Ameboporo (Ehambp)**	**X70851.1**	**212**	**AAGGAGAAATCCTCTGCAAC**	**CAAATAGCATTGGCATCAAC**

**Hypothetical protein 1 (Ehhypo-1)**	**XM_645356.2**	**114**	**CAAAAGCTTCAAGAAGAAGA**	**AATAACTTCTTCTTTCAA**
**Hypothetical Protein 6 (Ehhypo-6)**	**XP_00191351.1**	**118**	**GAGTCCAGGAGAACGACCAA**	**CCATCTCCACTCCTATTTGACA**
**Reference gene Actin-α (Ehactin-α)**	**XM_650064.2**	**211**	**AGCTGTTCTTTCATTATATGC**	**TTCTCTTTCAGCAGTAGTGGT**

Supplementary Table S2. Descriptive statistics of parasite gene expression

Gene	Post-exposure time (h)	n	Mean ± SD	Median (Q1-Q3)
Ehlect	1	4	2.10 ± 0.672	2.05 (1.46-2.85)
	3	4	2.96 ± 1.90	2.34 (1.41-5.74)
	12	2	3.87 ± 3.72	3.87 (1.24-6.50)
	24	4	3.43 ± 2.83	3.79 (2.55e-12-6.15)
Ehambp	1	4	142 ± 44.2	158 (78.0-174)
	3	4	95.2 ± 25.0	88.2 (73.5-131)
	12	2	44.0 ± 6.43	44.0 (39.4-48.5)
	24	4	8.05 ± 2.47	8.10 (5.50-10.5)
Ehcp2	1	4	35.1 ± 6.89	32.6 (30.1-45.3)
	3	4	4.38 ± 0.991	4.60 (3.03-530)
	12	2	2.00 ± 1.13	2.00 (1.20-2.80)
	24	4	0.383 ± 0.104	0.420 (0.230-0.460)
Ehcp5	1	5	1.70 ± 0.507	1.90 (1.15-2.15)
	3	5	1.33 ± 0.668	1.16 (0.50-2.30)
	12	3	0.393 ± 0.137	0.33 (0.30-0.55)
	24	5	0.274 ± 0.091	0.250 (0.17-0.41)
Ehnitrored	1	4	1.83 ± 0.468	1.72 (1.41-2.49)
	3	4	2.31 ± 139	1.90 (1.15-4.30)

	12	2	18.8 ± 5.44	18.8 (14.9-22.6)
	24	4	65.0 ± 15.1	68.5 (44.3-78.8)
Ehthiredox	1	4	3.17 ± 1.71	2.51 (1.97-5.70)
	3	4	3.21 ± 1.19	3.14 (1.87-4.70)
	12	2	2.24 ± 0.863	2.24 (1.63-2.85)
	24	4	46.5 ± 16.1	42.7 (32.0-68.6)
Ehhypo1	1	4	1.52 ± 0.805	1.68 (0.480-2.26)
	3	4	39.5 ± 13.5	40.0 (22.3-55.7)
	12	2	0.230 ± 0.085	0.230 (0.170-0.290)
	24	4	0.338 ± 0.126	0.325 (0.230-0.470)
Ehhypo3	1	4	0.372 ± 0.159	0.425 (0.140-0.500)
	3	4	0.397 ± 0.202	0.420 (0.130-0.620)
	12	2	0.625 ± 0.134	0.625 (0.530-0.720)
	24	4	0.660 ± 0.112	0.6 (0.540-0.790)
Ehhypo6	1	4	2.13 ± 0.270	2.09 (1.90-2.47)
	3	4	1.20 ± 0.264	1.11 (1.00-1.59)
	12	2	0.817 ± 0.259	0.817 (0.633-1.00)
	24	4	0.888 ± 0.132	0.866 (0.778-1.04)
Ehdovasa	1	4	0.535 ± 0.499	0.365 (0.160-1.25)
	3	4	46 ± 96.1	125 (52.1-280)
	12	2	4.50 ± 0.141	4.50 (4.40-4.60)
	24	4	2.46 ± 1.24	2.20 (1.26-4.19)

Mean ± SD and median (min-max) are provided for descriptive purposes regardless of data distribution.

Supplementary Table S3. Descriptive statistics of host gene expression

Gene	Post-exposure time (h)	n	Mean ± SD	Median (Min-Max)
tnf-α	1	4	0.389 ± 0.147	0.380 (0.220-0.574)

	3	4	1.93 ± 0.446	1.88 (1.51-2.46)
	12	3	1.57 ± 0.318	1.62 (1.23-1.86)
	24	4	1.91 ± 0.898	1.82 (1.10-2.88)
tnf-β	1	5	2.31 ± 1.43	2.0 (0.812-4.23)
	3	5	0.486 ± 0.17	0.53 (0.250-0.707)
	12	5	0.636 ± 0.149	0.61 (0.435-0.812)
	24	5	0.752 ± 0.355	0.75 (0.25-1.23)
il-8	1	4	0.784 ± 0.343	0.720 (0.467-1.23)
	3	4	0.531 ± 0.130	0.543 (0.380-0.660)
	12	3	0.627 ± 0.775	0.220 (0.140-1.52)
	24	4	1.44 ± 0.488	1.47 (0.812-2.00)
il-2	1	5	2.41 ± 0.801	2.30 (1.40-3.50)
	3	5	0.533 ± 0.126	0.50 (0.354-0.660)
	12	4	1.73 ± 0.642	1.63 (1.07-2.60)
	24	5	1.40 ± 0.594	1.40 (0.750-2.43)
il-4	1	4	0.314 ± 0.089	0.296 (0.230-0.435)
	3	4	1.72 ± 0.722	1.181 (0.812-246)
	12	3	1.58 ± 0.071	1.62 (1.50-1.63)
	24	4	2.03 ± 0.919	1.88 (1.10-3.25)
il-17	1	4	2.45 ± 0.433	2.38 (2.00-3.03)
	3	4	4.56 ± 1.82	4.45 (2.46-6.90)
	12	3	1.93 ± 0.700	2.14 (1.15-2.5)
	24	4	1.96 ± 0.397	2.02 (1.52-2.30)
il-10	1	4	0.222 ± 0.115	0.200 (0.109-0.380)
	3	4	0.339 ± 0.040	0.345 (0.287-0.379)
	12	3	1.03 ± 0.631	0.800 (0.540-1.74)
	24	4	2.40 ± 0.439	2.29 (2.00-3.03)
int- α	1	4	0.271 ± 0.160	0.227 (0.130-0.500)

3	4	1.21 ± 1.08	0.730 (0.570-2.82)
12	3	1.66 ± 1.29	2.14 (2.14-2.64)
24	4	2.32 ± 1.58	2.23 (0.520-4.30)

Mean ± SD and median (min-max) are provided for descriptive purposes regardless of data distribution.

Supplementary Table S4. Tukey and Games-Howell post hoc test comparing post-infection time points for *E. histolytica* genes showing significant differences in ANOVA analyses.

Gene	Post-infection time comparison	p-value	Test
Ehambp	1 h vs 3 h	0.115	Tukey
	1 h vs 24 h	<0.001	
	3 h vs 24 h	0.006	
Ehcp5	1 h vs 3 h	0.764	Games-Howell
	1 h vs 12 h	0.011	
	1 h vs 24 h	0.010	
	3 h vs 12 h	0.107	
	3 h vs 24 h	0.076	
	12 h vs 24 h	0.598	
Ehhypo6 (log10)	1 h vs 3 h	<0.001	Tukey
	1 h vs 24 h	<0.001	
	3 h vs 24 h	0.185	

Statistical significance was set at $p < 0.05$.

Supplementary Table S5. Dunn's post hoc test comparing post-infection time points for *E. histolytica* genes showing significant differences in Kruskal-Wallis analyses. P-values were adjusted using Bonferroni correction.

Gene	Post-infection time comparison	p (adjusted)
Ehcp2	1 h vs 3 h	0.350
	1 h vs 24 h	0.005

	3 h vs 24 h	0.350
Ehnitrored	1 h vs 3 h	1.000
	1 h vs 24 h	0.048
	3 h vs 24 h	0.063
Ehhypo1	1 h vs 3 h	0.348
	1 h vs 24 h	0.348
	3 h vs 24 h	0.005
Ehdovasa	1 h vs 3 h	0.005
	1 h vs 24 h	0.350
	3 h vs 24 h	0.350

Statistical significance was set at $p < 0.05$.

Supplementary Table S6. Tukey and Games-Howell post hoc tests comparing post-infection time points for host cytokine gene expression showing significant differences in ANOVA analyses.

Gene	Post-infection time comparison	p-value	Test
tnf-α	1 h vs 3 h	0.013	Games-Howell
	1 h vs 12 h	0.067	
	1 h vs 24 h	0.011	
	3 h vs 12 h	0.624	
	3 h vs 24 h	1.000	
	12 h vs 24 h	0.894	
tnf-β (log10)	1 h vs 3 h	0.002	Tukey
	1 h vs 12 h	0.013	
	1 h vs 24 h	0.020	
	3 h vs 12 h	0.782	
	3 h vs 24 h	0.657	
	12 h vs 24 h	0.996	
il-4	1 h vs 3 h	0.034	Games-Howell
	1 h vs 12 h	0.082	

	1 h vs 24 h	0.010	
	3 h vs 12 h	0.978	
	3 h vs 24 h	0.950	
	12 h vs 24 h	0.777	
il-2	1 h vs 3 h	<0.001	Tukey
	1 h vs 12 h	0.361	
	1 h vs 24 h	0.071	
	3 h vs 12 h	0.039	
	3 h vs 24 h	0.141	
	12 h vs 24 h	0.833	
il-17	1 h vs 3 h	0.063	Tukey
	1 h vs 12 h	0.913	
	1 h vs 24 h	0.912	
	3 h vs 12 h	0.031	
	3 h vs 24 h	0.021	
	12 h vs 24 h	1.000	
il-10 (log10)	1 h vs 3 h	0.288	Tukey
	1 h vs 12 h	0.002	
	1 h vs 24 h	<0.001	
	3 h vs 12 h	0.028	
	3 h vs 24 h	<0.001	
	12 h vs 24 h	0.033	

Statistical significance was set at $p < 0.05$.
